# Health care consumption and costs due to foot and ankle injuries in the Netherlands, 1986–2010

**DOI:** 10.1186/1471-2474-15-128

**Published:** 2014-04-12

**Authors:** A Siebe De Boer, Tim Schepers, Martien JM Panneman, Ed F Van Beeck, Esther MM Van Lieshout

**Affiliations:** 1 Trauma Research Unit Department of Surgery, Erasmus MC, University Medical Center Rotterdam, P.O. Box 2040, 3000 CA Rotterdam, The Netherlands; 2 Consumer & Safety Institute, P.O. Box 75169, 1070 AD Amsterdam, The Netherlands; 3 Department of Public Health, Erasmus MC, University Medical Center Rotterdam, P.O. Box 2040, 3000 CA Rotterdam, The Netherlands

**Keywords:** Ankle, Costs, Epidemiology, Foot, Health care use, Injuries

## Abstract

**Background:**

Foot and ankle injuries account for a large proportion of Emergency Department attendance. The aim of this study was to assess population-based trends in attendances due to foot and ankle injuries in the Netherlands since 1986, and to provide a detailed analysis of health care costs in these patients.

**Methods:**

Age- and gender-standardized emergency attendance rates and incidence rates for hospital admission were calculated for each year of the study. Injury cases and hospital length of stay were extracted from the National Injury Surveillance System (non-hospitalized patients) and the National Medical Registration (hospitalized patients). Data were grouped into osseous and ligamentous injuries for foot and ankle separately. An incidence-based cost model was applied to calculate associated direct health care costs.

**Results:**

Since 1986 the overall emergency attendance rate decreased from 858 to 640 per 100,000 person years. In non-admitted patients (90% of cases), ligamentous injuries approximately halved, whereas osseous injuries increased by 28% (foot) and 25% (ankle). The incidence rate for hospital admission increased by 35%, mainly due to an almost doubling of osseous injuries. Attendance rates showed a peak in adolescents and adults until ~45 years of age in males and (less pronounced) in females. The total number of hospital days decreased to 58,708 days in 2010. Hospital length of stay (HLOS) increased with age and was highest for osseous injuries. HLOS was unaffected by gender, apart for longer stay in elderly females with an osseous ankle injury. Health care costs per case were highest for osseous injuries of the ankle (€ 3,461). Costs were higher for females and increased with age to € 6,023 in elderly males and € 10,949 in elderly females. Main cost determinants were in-hospital care (56% of total costs), rehabilitation/nursing care (15%), and physical therapy (12%).

**Conclusions:**

Since 1986, the emergency attendance rate of foot and ankle injuries in the Netherlands decreased by 25%. Throughout the years, the attendance rate of (relatively simple) ligamentous injuries strongly reduced, whereas osseous injuries nearly doubled. Attendance rates and health care costs were gender- and age-related. Main cost determinants were in-hospital care, rehabilitation/nursing care, and physical therapy.

## Background

During the last decades, quality of trauma care (both prehospital and hospital care) has improved and complication rates have decreased [1,2]. Lower extremities are among the most frequently injured body regions in trauma patients [2-5]. The majority of foot and ankle injuries occur during sports or work; they form a leading cause of trauma hospitalizations [3-7]. As foot and ankle injuries account for over 20% of all injury patients visiting an Emergency Department (ED), research on trends in emergency attendance and health care use in this group is needed [8].

Population-based knowledge on emergency attendance rates, health care use and economic burden of foot and ankle injuries is essential for the allocation of health care services, optimization of preventive measures and research purposes, but it also provides a forecast for the future. Most epidemiologic studies on foot and ankle injuries focused on one distinct subgroup such as a specific type of injury, anatomical region, or age group [8-20]. Most studies used data from a single hospital or a regional database [8,9,12,15,16,18,19,21-23]. Some papers used a national injury database [10,11,17,20,24-26]. No papers summarize long-term population trends in emergency attendance rates, health care used and costs of all foot and ankle injuries presented to the emergency department at a national level. Detailed evaluations of costs, gaining insight in the parameters that contribute most to the overall costs, such as cost for hospital stay, physical therapy and rehabilitation are not available. Due to budgetary restraints and increasing health care costs, such economic analyses are gaining importance.

Therefore the aim of the current study was to examine long-term population-based trends in the emergency attendance and associated hospitalization and health care costs of foot and ankle injuries in the Netherlands from 1986 to 2010.

## Methods

### Data sources

For this retrospective study data were collected for patients with foot and ankle injuries in the Netherlands in the period 1986–2010. Injury cases were extracted from the National Injury Surveillance System (LIS) [27] and National Medical Registration (LMR) [28], to include non-hospitalized and hospitalized patients, respectively. LIS is a continuous monitoring system that records unintentional and intentional injuries. It has been implemented in 17 hospital EDs, resulting in a representative 12% sample of all injury-related ED visits in the Netherlands [27]. These hospitals are geographically distributed across the country with their adherence population being representative for the Dutch population in age and gender structure [29]. LMR collects data regarding hospital admissions, admission diagnosis, gender, age, and length of hospital stay. LMR is centrally evaluated for plausibility and completeness before entry into the LIS database [27]. LMR has almost complete national coverage (<5% missing except 12% for 2007) and figures are extrapolated to full national coverage for each year. An extrapolation factor was determined by comparing the adherence population of the participating hospitals with the total Dutch population in each year [28]. Patients are included in LIS and LMR according to their main diagnosis at discharge, which is generally the most severe injury. Coding of patients was consistently based upon full patient chart review including routine radiological assessment as available in the patient files.

Injuries in hospitalized patients (LMR) were defined using the International Classification of Diseases, 10^th^ revision (ICD-10, including codes for injuries to the lower leg (S82), foot (S92-93, S79), and ankle (S82, S93, S97) [30]. During the study period the ICD-version changed from the 9^th^ to the 10^th^ revision version in the year 2010. Data encoded using ICD-9 were extracted using a conversion table developed by the World Health Organization Collaborating Center for the Family of International Classifications (WHO FIC). Injuries in non-hospitalized patients (LIS) were defined using injury type descriptions. In order to report data on both databases combined (which is also the most clinically relevant grouping), patients were grouped into four injury categories; 1) Osseous ankle injuries; 2) Ligamentous ankle injuries; 3) Osseous foot injuries; 4) Ligamentous foot injuries (Table 1). Since the LIS database contains a limited number of injury classes, a more detailed analysis was not possible.

**Table 1:** Subdivision of the ICD-codes from the LMR database and the injury types from the LIS database in the four main injury groups

	**LMR database**	**LIS database**
**Foot injuries**		
**Osseous**	Fracture of calcaneus (S920)	Fracture of foot/toe
	Fracture of talus (S921)	Dislocation of foot/toe
	Fracture of other tarsal bone(s) (S922)	
	Fracture of metatarsal bone (S923)	
	Fracture of other toe (S925)	
	Fracture of foot, unspecified (S929)	
	Dislocation of toe(s) (S931)	
	Dislocation of other and unspecified parts of foot (S933)	
Ligamentous	Sprain and strain of toe(s) (S935)	Sprain and strain foot/toe
	Sprain and strain of other and unspecified parts of foot (S936)	Muscle-/tendon injury foot/toe
**Ankle injuries**		
**Osseous**	Fracture of fibula alone (S824)	Fracture of ankle
	Fracture of medial malleolus (S825)	Dislocation of ankle
	Fracture of lateral malleolus (S826)	
	Fractures of other parts of lower leg (S828)	
	Dislocation of ankle joint (S930)	
Ligamentous	Sprain and strain of ankle (incl. Achilles tendon rupture) (S934)	Muscle-/tendon injury of ankle
		Sprain and strain of ankle
		Achilles tendon injury

Data regarding hospital length of stay (HLOS) were extracted from the LMR database for 10-year age categories. In order to assess trends in HLOS over time, the mean HLOS was averaged over 5-year intervals from 1991–2010. The time periods for the different analyses (1986–2010 for incidence rates, 1991–2010 for HLOS, and 2010 for health care consumption and associated costs) were based on data availability.

The study was exempted by the local Medical Research Ethics Committee Erasmus MC (No. MEC-2014-006).

### Calculation of emergency attendance rates and incidence rates for hospital admission

Data were analyzed using the Statistical Package for the Social Sciences (SPSS) version 16.0 for Windows.

Age-specific emergence attendance rates (for all patients presented to the ED) and incidence rates for hospital admission (for all patients admitted to hospital) were calculated in 5-year age groups. This was done for the total population and for males and females separately. For each age group the absolute numbers of hospitalized and non-hospitalized cases with foot and ankle injuries were extracted from the LMR and LIS database, respectively. Since patient numbers in the LIS database were obtained from a sample, they were weighted in order to create national estimates. An extrapolation factor was determined by comparing the number of admitted injury patients in the LIS database with the total number of admitted injury patients in the LMR database. In order to adjust for differences in the demographic composition over time, emergency attendance rates and incidence rates for hospital admission were standardized for age (in 5-year age groups) and gender using a direct standardization method. The age- and gender-specific emergency attendance rates and incidence rates for hospital admission per 100,000 person years were calculated based upon the Dutch mid-year standard population. Mid-year population sizes for all age groups were obtained from Statistics Netherlands [31]. Age-adjusted emergency attendance rates and incidence rates for hospital admission were calculated using “direct standardization” [32]. The average number of persons in each 5-year age class for each year of the study (1986–2010) was calculated. This number was used as the standard (reference) population, as described previously [33,34]. Overall increase in hospital admissions was calculated for 2010 in per cents relative to 1986.

### Calculation of costs

The incidence-based Dutch Burden of Injury Model, which has been used in ten European countries, was used in order to measure and describe the health care costs for the year 2010 [24,33,35-38]. Patient numbers, health care consumption, and related costs were calculated for the four injury groups using the LIS database, the National Hospital Discharge Registry, and a patient follow-up survey to calculate associated direct health care costs in 2010. The patient follow-up survey collected data on in-hospital care, outpatient visits, general practitioner (G.P.) visits, outpatient physical therapy, home care, medication, and aids and appliances [29]. Costs and health care consumption are injury-, gender- and age-dependent. In our model, the age- and injury-specific costs were based upon the estimated health care supplied to the individual patients. Costs were determined for the following categories: 1) ambulance care; 2) G.P. visits; 3) in-hospital care; 4) home care; 5) rehabilitation and nursing home care; and 6) physical therapy. Health care costs of injuries were calculated by multiplying incidence, health care volumes (*e.g.*, length of stay in hospital or institution, the number of outpatient visits, G.P. visits, home care hours, and physical therapy treatments) with unit costs (*e.g.*, costs per day in hospital). Unit costs were estimated according to national guidelines for health care costing [38]. Age-specific costs are presented in 10-year age groups for men and women separately.

## Results

### Emergency attendances and hospital admissions

During the study period, the absolute number of patients reporting to an ED with a foot or ankle injury decreased from 124,595 in 1986 to 106,157 in 2010. The emergency attendance rate of all injuries combined decreased from 858 to 640 per 100,000 persons (-25.4%). Whereas ligamentous injuries approximately halved, osseous injuries nearly doubled.

**Figure 1:**
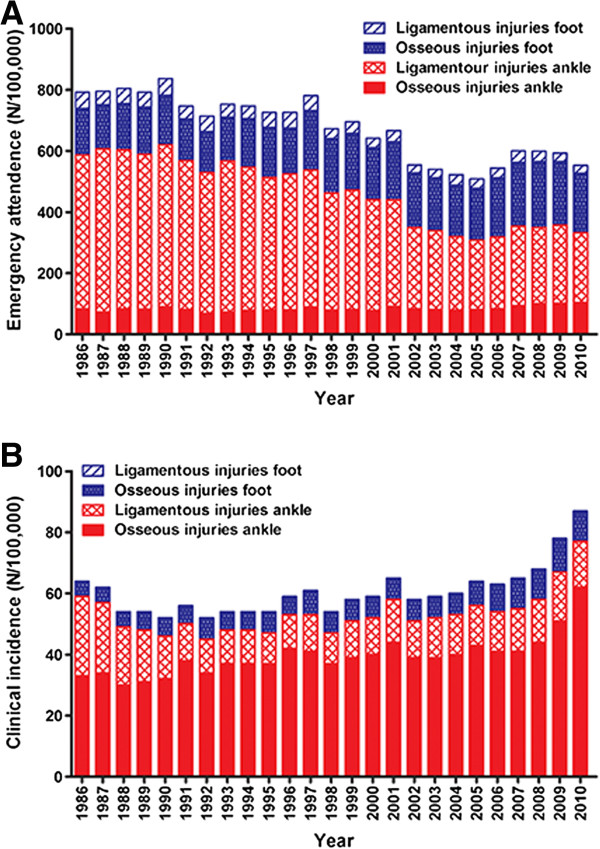
**Trends in age- and gender adjusted emergency attendance rates and incidence rates for hospital admission (per 100,000 person years) of foot and ankle injuries in the period 1986–2010 for non-admitted (A) and admitted (B) patients.** Emergency attendance refers to all patients presented to the Emergency Department, and incidence rates for hospital admission refers to all patients admitted to hospital.

In non-admitted patients, representing 90% of patients, the overall emergency attendance rate decreased by 30.2% (Figure 1A). This was mainly due to a decrease in ligamentous injuries of the ankle (504/100,000 in 1986 versus 228/100,000 in 2010; -54.8%) and foot (26/100,000 in 2010; -50.9%). Osseous injuries in the foot and ankle, however, increased by 28.3% (152/100,000 in 2010) and 25.3% (104/100,000 in 2010), respectively.

The admission rate increased from 7.6% in 1986 to 13.8% in 2010 (Figure 1B). This was mainly due to a 31.8% admission rate of patients with osseous ankle injuries. Admission of patients with osseous foot injuries (4.0% admitted) or ligamentous injuries in the ankle (3.8%) or foot (<0.1%) was low. Since 1986, the incidence rate for hospital admission due to foot and ankle injuries increased by 35.4%. This was mostly due to increased incidences of osseous ankle injuries (33/100,000 in 1986 versus 62/100,000 in 2010; +87.9%). The incidence rate for hospital admission due to ligamentous ankle injuries diminished with 42.3% (26 to 15/100,000).

**Figure 2:**
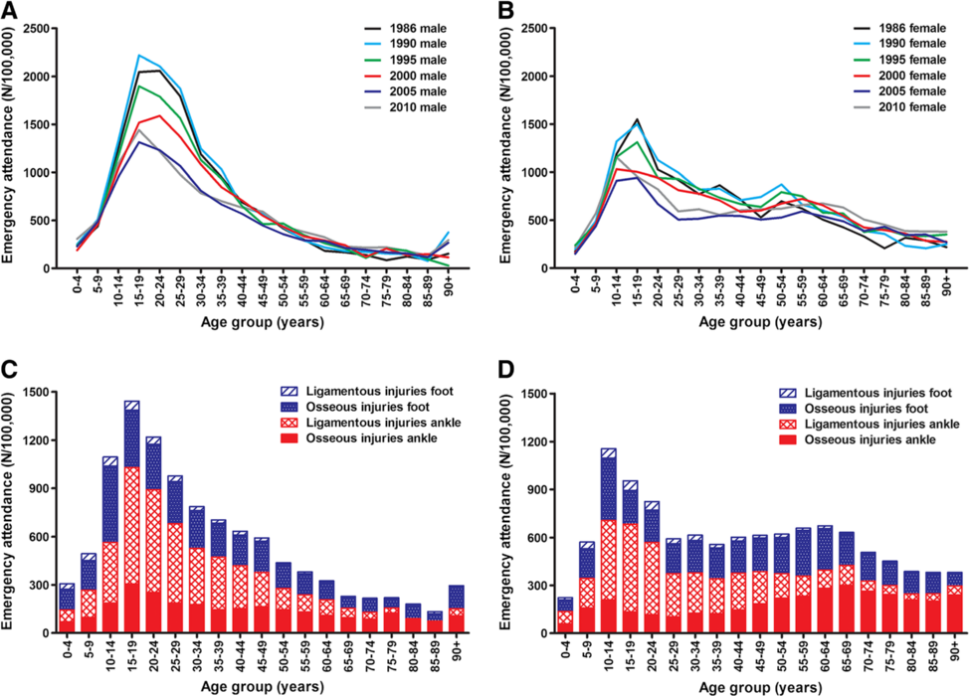
**Trends in emergency attendance (per 100,000 person years) of foot and ankle injuries by age.** The upper panels show data for six different years for males **(A)** and females **(B)**. In the lower panels, data are separated into osseous and ligamentous injuries of the foot and ankle. Data are shown for males **(C)** and females **(D)** in 2010.

The emergency attendance rates of foot and ankle injuries varied with age in males and, less pronounced, also in females (Figure 2A and 2B). Attendance rates showed a peak in adolescents and adults until ~45 years of age. Until this age the attendance rate in males was higher than in females. Since 1986 this peak in attendance has decreased in both genders. The decrease in incidence peaks at younger ages over time suggests a shift towards a higher mean age. Indeed, the mean age of patients increased throughout the study period by 8.3 years for osseous ankle injuries (from 32.1 ± 19.7 (SD) years in 1986 to 40.4 ± 22.7 in 2010) and by 4.0 years for osseous foot injuries (from 31.6 ± 18.5 in 1986 to 35.5 ± 20.9 in 2010), Mean ages were much more stable for ligamentous injuries (age increased from 25.4 ± 15.7 to 26.7 ± 17.9 years for the foot and from 27.0 ± 13.4 to 29.6 ± 16.7 years for the ankle).

Figure 2C and 2D show age-trends of the four main injury types in 2010. Again, a peak in adolescents was seen, especially in males. Whereas the incidence in all injury types reduced with age in males, the incidence of osseous injuries in elderly women remained more stable.

### Hospital length of stay

**Figure 3:**
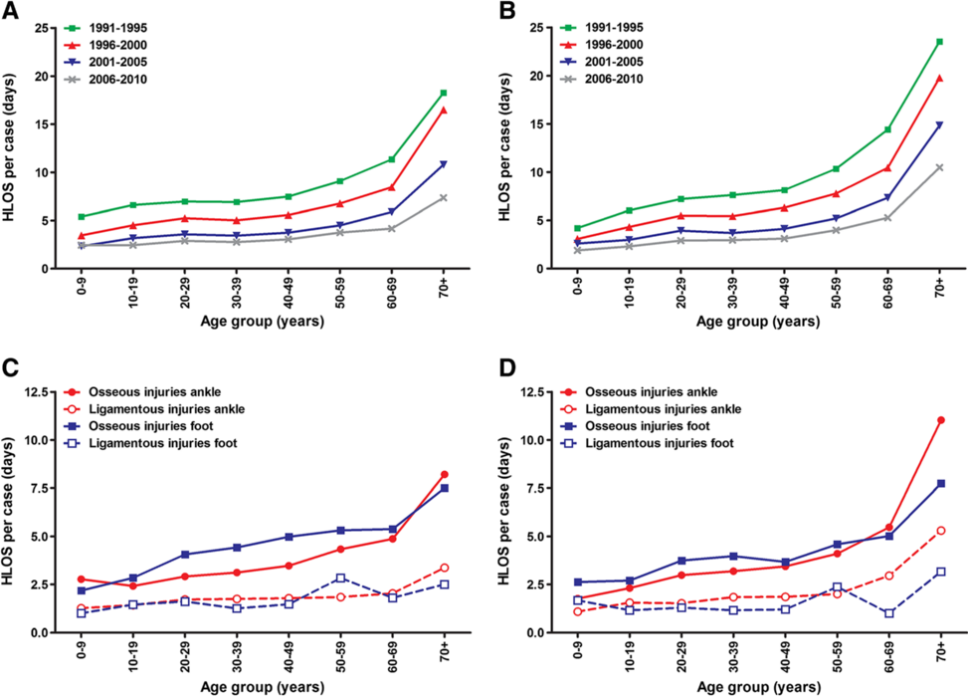
**Age-related trends in hospital length of stay due to foot and ankle injuries.** The upper panels show data for four different time periods for males **(A)** and females **(B)**. The lower panels show data for the four main categories of injuries in males **(C)** and females **(D)** in 2010.

Hospital length of stay (HLOS) in four consecutive five-year periods is shown in Figure 3A and 3B. Each period showed a gradual increase with age, yet over time the HLOS decreased for all age groups. The HLOS per case more than halved both in males (7.8 days in 1991 versus 3.3 in 2010) and females (11.5 days versus 4.9). The total number of hospital days for men and women of all ages combined decreased from 78,951 days in 1991 to 58,708 days in 2010. Patients aged 20–65 year accounted for 51% of all hospital days.

The HLOS for different types of injuries is shown in Figure 3C and 3D (for males and females, respectively). Osseous injuries caused the longest hospital stay per case in almost every age group, with limited differences between foot and ankle injuries. HLOS in males and females was similar for all injury types; in every age group the difference was restricted to one day at most. Exceptions to this were noted in elderly (70+) with an ankle injury; HLOS for osseous ankle injuries was 11.0 days in females versus 8.2 days in males. HLOS for ligamentous ankle injuries was 5.3 and 3.4 days, respectively.

### Costs for health care consumption

The overall costs for all patients amounted 161.9 million euro in 2010. Between 2001 and 2010, costs remained fairly stable; an overall increase of 1.2% was noted (data not shown).

Costs per case for all injuries and age groups combined were € 1,802 for females and € 1,204 for males (Table 2). For all four injury groups, the costs per case were higher in females than in males; they were highest for osseous injuries of the ankle (€ 4,294 and € 2,549 in females and males, respectively) and the foot (€ 1,229 and € 898), and were lowest for ligamentous foot injuries (€ 740 and € 653).

**Table 2:** Total and mean cost of all injuries of the foot and ankle for admitted and non-admitted patients (2010)

	**Overall (males + females)**		**Males**		**Females**	
	**N cases**	**Cost per case (€)**	**N cases**	**Cost per case (€)**	**N cases**	**Cost per case (€)**
**Admitted patients**						
Osseous injuries foot	1,527	6,088 (1,469)	969	4,475 (961)	558	8,887 (2,349)
Ligamentous injuries foot	36	2,582 (199)	22	2,746 (244)	14	2,322 (129)
Osseous injuries ankle	8,737	7,383 (1,620)	3,931	5,415 (998)	4,806	8,993 (2,128)
Ligamentous injuries ankle	2,869	2,780 (281)	2,153	2,586 (251)	716	3,364 (373)
**Subtotal**	**13,169**	**6,217 (1,307)**	**7,075**	**4,417 (764)**	**6,094**	**8,306 (1,937)**
**Non-admitted patients**						
Osseous injuries foot	33,511	836 (126)	16,418	687 (93)	17,092	979 (157)
Ligamentous injuries foot	5,203	685 (96)	2,502	634 (85)	2,701	732 (106)
Osseous injuries ankle	15,916	1,308 (154)	7,832	1,110 (129)	8,084	1,500 (177)
Ligamentous injuries ankle	40,368	684 (82)	21,460	642 (69)	18,908	731 (97)
**Subtotal**	**94,998**	**842 (110)**	**48,213**	**733 (88)**	**46,785**	**955 (133)**
**All patients**						
Osseous injuries foot	35,038	1,065 (184)	17,387	898 (141)	17,650	1,229 (226)
Ligamentous injuries foot	5,240	698 (97)	2,525	653 (87)	2,715	740 (106)
Osseous injuries ankle	24,653	3,461 (673)	11,763	2,549 (420)	12,890	4,294 (904)
Ligamentous injuries ankle	43,237	823 (92)	23,613	819 (86)	19,624	827 (107)
**Total**	**108,167**	**1,496 (255)**	**55,288**	**1,204 (174)**	**52,879**	**1,802 (338)**

Mean costs per case are given, with the standard deviation between brackets.

**Figure 4:**
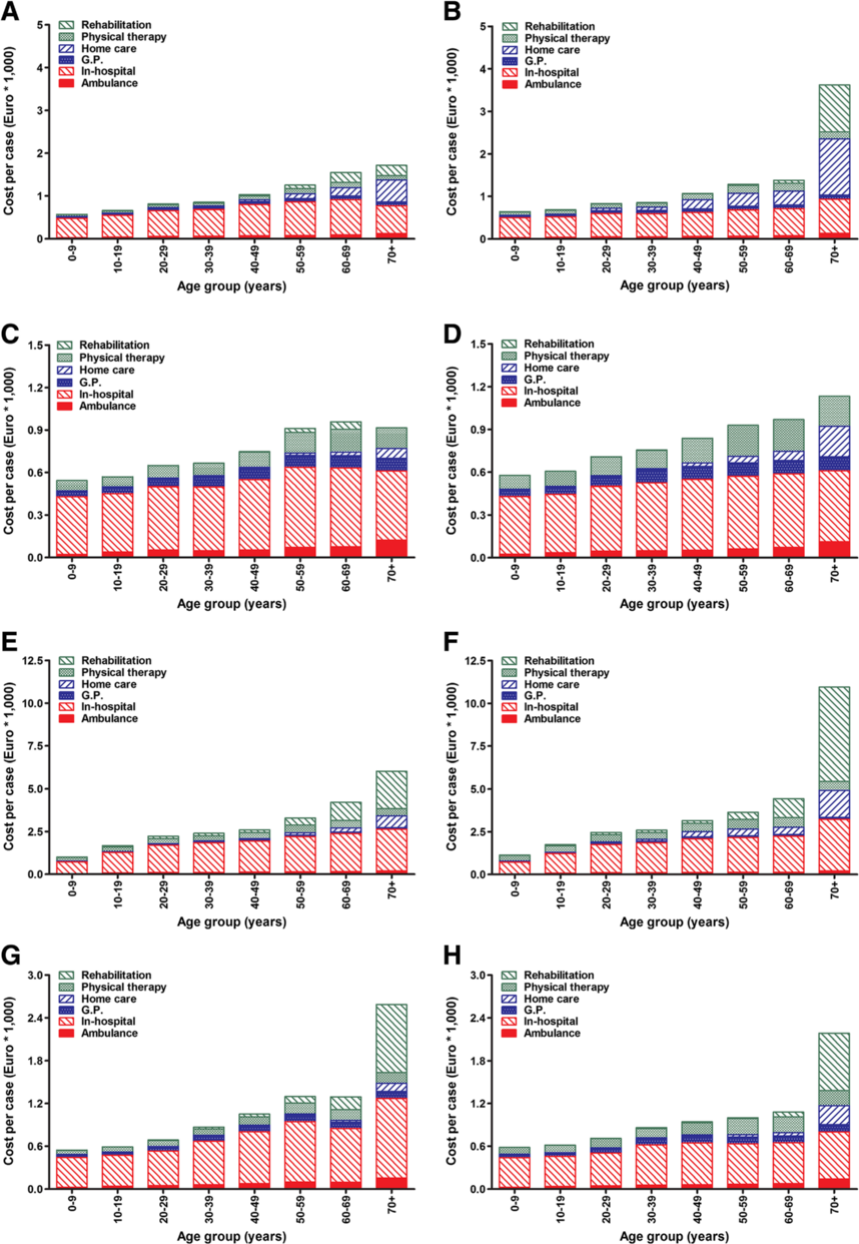
**Age- and injury-related costs per case for the treatment of foot and ankle injuries in males (A, C, E, and G) and females (B, D, F, and H), separated into different cost determinants.** Costs per case are given for osseous foot injuries **(A and B)**, ligamentous foot injuries **(C and D)**, osseous ankle injuries **(E and F)** and ligamentous ankle injuries **(G and H)**. Costs are shown separately for ambulance care, in-hospital care, general practitioner visits, home care, physical therapy, and rehabilitation/nursing home care. Data for 2010 are shown for admitted and non-admitted patients combined.

Figure 4 shows the costs per case for the four main injury groups, separated into costs for different types of health care use. In addition to costs per case being higher in females than in males, costs consistently increased with age for all four injury categories; from 0 to 70+ years, costs for all injuries combined increased 9.2-fold in females and 5.7-fold in males. The largest increase with age was seen for osseous injuries of the ankle (from € 996 to € 6,023 in males and from € 1,127 to € 10,949 in females; Figures 4E and F) and foot (from € 571 to € 1,716 in males and from € 642 to € 3,626 in females; Figures 4A and B). Ligamentous foot injuries showed only a 1.7-fold and 2.0-fold increase across the age groups in males and females, respectively (Figures 4C and D). Costs for ligamentous injuries were independent of gender. For osseous injuries, the costs per case were similar in males and females until 60 years of age, but a clear gender-dependency was noted for the 70+ group.

Costs for in-hospital care consistently contributed most to the total cost per case (64-69% of total costs in males and 45-62% in females; Figure 4). Physical therapy was the second largest determinant (8-19% of total costs). Ambulance care and G.P. visits each contributed less than 10% to the overall cost per case; they doubled or tripled over the age groups, but were unrelated to injury type and gender. For osseous foot and ankle injuries, the increase in costs over the age groups was mainly due to increased use of rehabilitation/nursing care and home care in the elderly. This effect was more pronounced in females than in males. The age-effect on costs in ligamentous foot injuries was mainly attributable to increased costs for physical therapy, G.P. visits, and home care. Higher use of home care also explained the larger cost increase in females. For ligamentous ankle injuries, in-hospital costs increased more with age in males, whereas costs for home care and physical therapy increased more in females.

## Discussion

Since 1986 the emergency attendance rate of ligamentous foot and ankle injuries consistently decreased, yet osseous injuries increased over time. Osseous injuries were the most expensive type of injury. The main cost determinants were in-hospital care and physical therapy.

This study shows the reduction in emergency attendance over time is mainly attributable to a decrease in clinically observed ligamentous ankle injuries. During the study period new guidelines and After Hours Medical Clinics were established [39,40], so patients with minor injuries can nowadays get G.P. consultations and treatment 24/7. The annual number of patients visiting a G.P. with distal lower extremity problems increased from 300,000 in 2000 to 600,000 in 2005 [39]. LIS and LMR do not record patients visiting only a G.P., so they were not included in our study.

The incidence rate of patients with osseous injuries strongly increased since 1986, especially in admitted patients. This may indicate a shift towards more complex injuries over time or an increase in the number of surgically treated fractures which is especially seen in ankle fractures [20,41,42]. Similar trends towards increased operative treatment have also been reported for other injuries [43-45].

Several studies have shown increased incidence rates of foot and ankle injuries in the last decades [2,8,41]. This is in line with an increase in fractures found in the current study, although we noted a decrease in emergency attendance overall. The profile and presentation of emergency department injuries have altered and the increase in osseous injuries may be proportional to the increase seen in all lower limb injuries.

Over the years, the HLOS per case decreased. Our data do not allow us to conclude whether that was due to improved health care programs, operative procedures and implants, or changes in admission and discharge guidelines. Introduction of evaluation guidelines like the Ottawa Ankle Rules [40] may also have resulted in earlier diagnosis and subsequent earlier treatment and lower complication rates. The increase in HLOS in elderly women with osseous injuries suggests a role for osteoporosis, as also noted before [8]. Osteoporotic fractures are often more complicated to treat, resulting in prolonged hospital stay.

One study reported on costs of foot and ankle fractures in the Netherlands in 1999, using the same cost model [24]. After correction for inflation, the corresponding costs in 2010 would be € 25.7 million (€ 861/case) for foot/toe fractures and € 54.5 million (€ 2,870/case) for ankle fractures. The higher costs observed in the current study may be attributable, at least partly, to more fractures treated operatively and higher costs for novel implants. Also, improvement in data sources on home and nursing care and on operative interventions may have resulted in more accurate, most likely higher, estimates of costs in the current study.

As expected, in-hospital care (especially admission days), rehabilitation/nursing care, and physical therapy were the main cost drivers. Similar results have been reported for ankle fractures [46]. In-hospital cost for osseous ankle fractures cost €4,000/case in our study, which was in line with data from Murray *et al.* reported £4730 (*i.e.*, €4230) [47]. The fact that the age effect was larger in females than in males may reflect that females tend to outlive their partners; elderly are more prone to losing their independence after sustaining a foot or ankle injury. Higher costs for osseous injuries were mainly attributable to longer HLOS.

This study is unique as it is a population-based study showing national and long-term trends in emergency attendance and hospitalization of patients with all foot and ankle injuries. Detailed data on health care costs is also novel. Most studies on lower leg injuries were restricted to one distinct injury or age group [8-19], focused on few hospitals, or were limited to (non-)hospitalized patients. Some studies used national injury databases [10,11,17,20,24-26]. National registry data more reliably represent the true health care problem than extrapolating data from one trial or hospital [27]. Although LIS-data covers 12% of the Dutch population, international validation studies have shown that the mathematical model underlying the extrapolation has a high level of completeness and validity [27]. Agreement of LIS recordings with hospital discharge systems and actual incidence of hospital admissions is high [27,48]. Both rural and urban areas and all levels of trauma care are included, supporting validity and generalizability of our findings.

We also acknowledge limitations, the most obvious being that patients who only visited a G.P./sports physician were not included. Although this indicates an underestimation of the problem at large, it can be expected that the excluded patients had minor injuries not requiring substantial treatment. Furthermore, there may be some statistical uncertainty due to underreporting of combined injuries, as patients are recorded based upon their main injury at discharge. A related limitation is that despite the introduction of evaluation guidelines like the Ottawa Ankle Rules [40], 8-18% of all foot fractures and 3-22% of ankle fractures are still missed at initial evaluation [49]. This likely caused a bias towards a lower emergency attendance rate, but this applies to all studies. A final limitation is that indirect health care costs like absenteeism and work disability were not taken into account in the cost model. Since the majority of patients with foot or ankle injuries are 20–60 years, the total societal burden will be higher than our data indicate. For calcaneal fractures, the work absenteeism costs exceeded the direct medical costs [50].

## Conclusions

The overall emergency attendance rate of foot or ankle injuries in the Netherlands seems to have decreased by 25% since 1986. The highest attendance was noted in patients aged 20–50 years. Whereas an approximately 50% reduction in ligamentous injuries was noted, the osseous injuries increased over time (25-28% in non-admitted patients, 87-100% in admitted patients), which might indicate a shift towards more substantial injuries. Attendance rates and health care costs were gender- and age-related. The main cost determinants were in-hospital care, rehabilitation/nursing care, and physical therapy.

## Abbreviations

ED: Emergency department; G.P.: General practitioner; HLOS: Hospital length of stay; ICD-10: International classification of diseases, 10^th^ revision; LIS: National injury surveillance system; LMR: National medical registration.

## Competing interests

The authors declare that they have no competing interests.

## Authors’ contributions

ASDB participated in data analysis and interpretation of data and assisted in drafting the manuscript. TS participated in the design of the study, interpreted the data, and critically revised the manuscript. MJMP collected the data from the databases, assisted in statistical analysis, interpreted the data, and critically revised the manuscript. EFVB interpreted the data and critically revised the manuscript. EMMVL designed the study, analyzed and interpreted the data, and drafted the manuscript. All authors read and approved the final manuscript.

## Pre-publication history

The pre-publication history for this paper can be accessed here:

http://www.biomedcentral.com/1471-2474/15/128/prepub
